# Panel evaluation of esthetic result after chest masculinization: Reliability and comparison with patient-reported satisfaction

**DOI:** 10.1016/j.jpra.2025.11.018

**Published:** 2025-11-22

**Authors:** Mirjam Saarinen, Sinikka Suominen, Maija Kolehmainen, Kaisu Ojala

**Affiliations:** Department of Plastic Surgery, Helsinki University Hospital and University of Helsinki, Helsinki, Finland

**Keywords:** Transgender men, Chest masculinization, Top surgery, Aesthetic outcome, Panel evaluation, BODY-Q Chest Module

## Abstract

Chest masculinization is the most common surgical intervention in transgender men. Patient-reported satisfaction is generally good, but we lack other tools for evaluation of the postoperative result and knowledge about the surgeons understanding of a desirable result. Panel evaluation of the esthetic result has been used widely in other surgical areas. However, regarding chest masculinization, the use of panel evaluation is limited. This study aims to evaluate esthetic postoperative result after chest masculinization by an expert panel and to assess the reliability of panel evaluation. Furthermore, we aim to compare the panel evaluation with patient-reported satisfaction. The data comprised all patients receiving chest masculinization from 2005 to 2018 with sufficient postoperative images. Sufficient images were found for 48 patients, 26 of whom filled in the patient-reported outcome tool BODY-Q Chest Module. The esthetic result was evaluated by a six-person expert panel. On a scale from 1 (worst) to 4 (best), panelists’ overall score yielded a median of 3.2. Inter-rater reliability was low. Scores given by female and male panelists did not differ significantly. When evaluating items scored by both the panelists and the patients, no significant correlation emerged. We conclude that the esthetic result after chest masculinization is generally good. However, even professionals lack a consensus on the desired result. The panel evaluation in this study proved to be unreliable, but due to biases in patient-reported outcomes, we call for the development of a validated clinician-reported tool for assessment of esthetic result after chest masculinization.

## Introduction

The demand for gender-affirming surgery is increasing. Chest masculinization is usually the first and often the only surgical intervention performed on transgender men. The patients expect relief from gender dysphoria and social acceptance, while surgeons aim for a natural masculine outcome and pay attention to technical issues like scar burden and scar placement. The understanding of the desirable result might therefore differ between the operating surgeon and the patient. To better cater to the patients’ needs, we wanted to evaluate if the health care professionals and patients have a different perspective on the results.

Several methods have been developed to assess postoperative esthetic results. Only recently has there been patient-reported outcome instruments developed and validated to measure patient-reported outcome after chest masculinization in transgender men.^1^, ^2^, ^3^ Patient-reported outcome measures (PROM) are known to prioritize the patients’ needs and enable standardized monitoring over time. However, limitations and biases include suspicion towards healthcare providers and their motives, redundant information, and inaccuracy in estimating problems.^4^^,^ ^5^ Therefore, using PROM as the only outcome tool in clinical practice might not be advisable.

For evaluation of postoperative esthetic result, panel evaluation has been widely used in several surgical areas and has in some circumstances been shown to be a reliable method for esthetic evaluation.^6^^,^ ^7^ However, reliability depends on the tool.^8^ The optimal size of a panel is under debate but has been suggested to be three to four people.^9^, ^10^, ^11^ The composition of the panel is also known to affect the results, and therefore, a gender-balanced panel with various professionals is often favored.^10^^,^ ^12^^,^ ^13^

Tools for clinician-reported esthetic evaluation have been developed but focus on a feminine result.^9^^,^ ^14^ Meanwhile, neither validation of panel evaluation nor development of a structured tool for clinician-reported outcome after chest masculinization has been accomplished. Instead, authors apply self-designed items often on Likert scales or modify tools developed for other target groups.^15^, ^16^, ^17^, ^18^ Despite the insufficiency of knowledge in the area concerning masculinizing chest surgery, in breast-conserving therapy (BCT) a four-point scale has been suggested as suitable for panel evaluation.^10^

Software programs have been developed for objective assessment of esthetic result in other areas of surgery such as oncoplastic breast surgery.^19^ These tools are, however, not applicable for masculinizing chest surgery, and we lack objective tools for evaluation of masculine chest outcomes.

Since surgeons aim to provide the best possible esthetic and functional result, their opinion on the optimal outcome plays a crucial role in the process. Other studies on esthetic outcome have reported disparity between the evaluations made by surgeons and patients and this warrants attention to enhance understanding and communication between the parties.^14^^,^ ^20^

This study aimed to evaluate the reliability of panel evaluation of postoperative esthetic result after chest masculinization in transgender men and to compare the esthetic evaluation with patient-reported satisfaction. Our intent was to add to the data on postoperative esthetic result and raise awareness of the benefits and limitations of different evaluation modalities.

## Materials and methods

### Patients

A retrospective patient material, containing all 225 transgender men receiving chest masculinization at Helsinki University Hospital (HUS) between January 1, 2005 and December 31, 2018, was collected from patient records. Altogether 225 patients were identified using the International Classification of Diseases (ICD)-10 code F64.0 for transsexualism combined with procedure codes for chest contouring surgery*.* The transition process followed the Finnish national guideline on treatment of gender dysphoria in transgender people.^21^ In Finland, a psychiatric assessment is required by law prior to gender affirming surgery. All patients were referred from a psychiatric transgender unit, where they undergo thorough psychiatric evaluation before receiving the diagnosis F64.0*.* Non-binary patients were not included in the study. In Finland, gender affirmation surgery is included in public healthcare and requires by law patient age of 18 years. Twelve months of masculinizing hormonal treatment has been used in our clinic as a general requirement for masculinizing chest surgery. The STROBE guidelines were followed.

### Panel evaluation of esthetic result

Postoperative images were collected from clinical databases. For 48 patients, sufficient images comprising one image directly from the front with upright position, one image directly from the front with forward-leaning position, one image from the left side with upright position, and one image from the right side with upright position were found. The images were taken at least 12 months after the primary operation or, in case of secondary corrections, the images were taken at least 6 months after the last correction.

**Figure 1:**
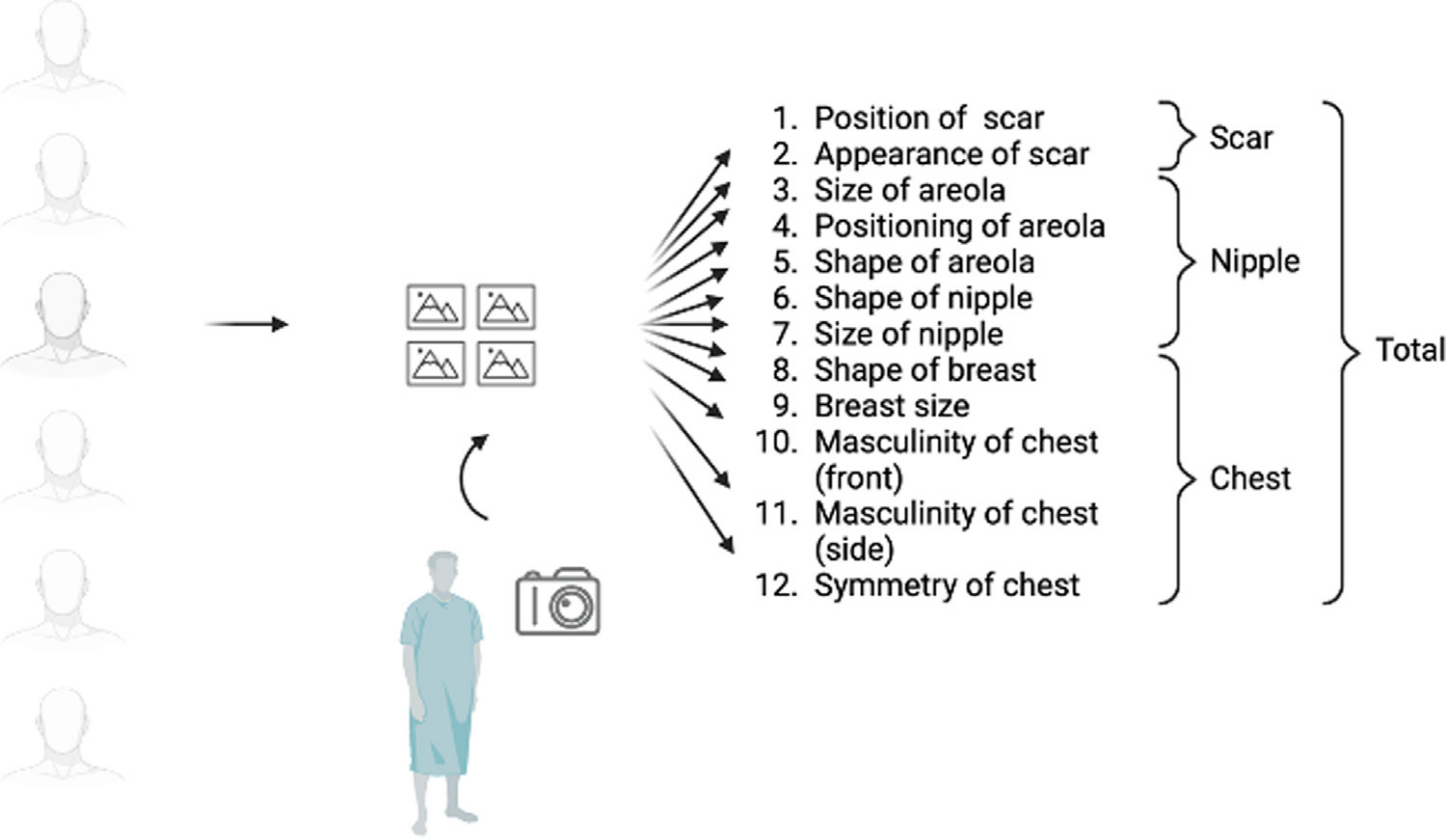
Illustration of the panel evaluation and division of separate items into scar-, nipple-, and chest-specific items. Created with BioRender.com.

The images were evaluated by a panel consisting of four plastic surgeons (two female, two male), one licensed physician (female), and one registered nurse (male). The plastic surgeons were not involved in operating on the evaluated patients. The panelists were requested to evaluate 12 author-designed aspects in the images on a four-point Likert scale (poor – satisfactory – good – very good). The aspect were listed as follows: one, positioning of the scar; two, appearance of the scar; three, size of the areola; four, positioning of the areola; five, shape of the areola; six, shape of the nipple; seven, size of the nipple; eight, shape of the breast; nine, size of the breast; ten, masculinity of the chest from the front; 11, masculinity of the chest from the side; 12, symmetry of the chest. An overall grade was calculated as the mean of the separate aspects. The process is illustrated in Figure 1.

### Patient-reported outcome

The patients were invited by letter to participate in a retrospective cross-sectional survey that comprised an evaluation of the patient-reported outcome after surgical chest masculinization. One patient (*n* = 1) could not be approached due to unknown address. The invitation package contained a general information letter, an informed consent form, the BODY-Q Chest Module, and a prepaid return envelope. The initial invitations were sent in May, 2020, and those who did not reply received a second invitation in September, 2020.

The BODY-Q Chest Module is the only PRO instrument designed and validated for transgender men undergoing chest surgery available in Finnish.^1^^,^ ^22^^,^ ^23^ It comprises a chest scale (ten items on appearance and one standalone item on scar appearance) and a nipple scale (five items). In addition, the chest scale contains one optional item evaluating scars in case of a previous surgery. Each item is assessed on a scale from one to four (very dissatisfied - somewhat dissatisfied - somewhat satisfied - very satisfied). The item scores are converted into a single score according to the instructions of the instrument, providing a score with range 1–100 and 0–100 for the chest and nipple scales, respectively.

### Statistics

Statistical analyses were conducted using RStudio version 2023.12.0 + 369 with packages “lpSolve”, “devtools”, “irr”, “dplyr”, and “ggplot2”. Data are reported as medians and interquartile ranges (IQR) for non-normal variables, means and standard deviations (SD) for normal variables, counts (N), and percentages (%). Normality of data was evaluated with the Shapiro-Wilk test.

Inter-rater reliability for the panel was evaluated per question with the intraclass correlation coefficient (ICC) using a two-way random effects model (type = consistency, unit = single). The reliability was interpreted from the ICC estimate as poor (<0.5), moderate (0.5–0.75), good (0.75–0.9), or excellent (>0.9).^24^

An overall grade was calculated for each panelist as a mean of the separate items.

To evaluate difference between the score given by male and female panelists, the overall grades given by the panelists were compared with Wilcoxon signed-rank test.

Due to the numerous statistical analyses, the level of statistical significance was defined after Bonferroni correction.

Single items included in the BODY-Q Chest Module and evaluated by the expert panel were analyzed for correlation. The correlation between the panel and participants was estimated with Kendall rank correlation and differences between groups with Kruskal-Wallis test.

## Results

**Figure 2:**
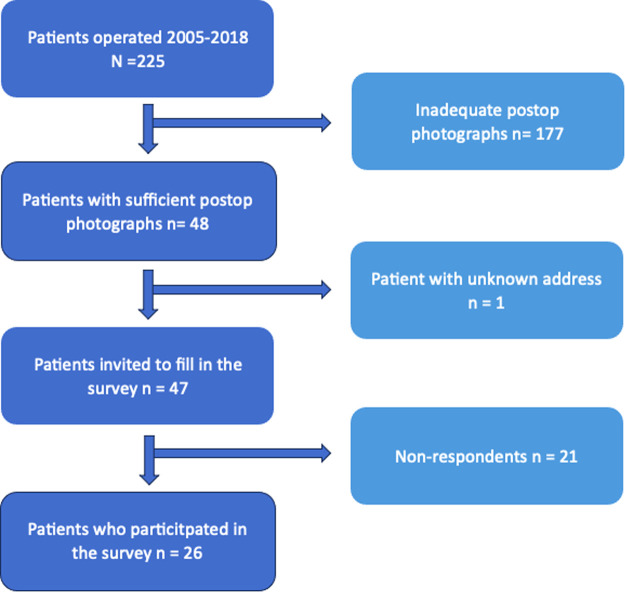
Flow-chart of patient selection.

The selection of the study population is visualized in Figure 2. Forty-eight patients with sufficient postoperative photographs were identified. Of these patients, 26 filled in the BODY-Q Chest Module (54 %). Clinical features of the patients are shown in Table 1. The threshold for statistical significance was set at 0.05/30 = 0.0017.

**Table 1:** Clinical features.

	Patients *N* = 48
Age at surgery, median (IQR)	24 (IQR = 6)
Surgical technique, n (%) • Periareolar techniques • Horizontal incision with pedicular NAC relocation • Horizontal incision with free NAC graft • Horizontal incision with other or mixed NAC technique	29 (60 %) 10 (21 %) 7 (15 %) 2 (4 %)

IQR, interquartile range; NAC, nipple-areolar complex.

The median overall score of all items given by the panel was 3.2 (IQR 0.5). The median overall score in chest-specific items was 3.4 (IQR 0.6) and in nipple-specific items 3.2 (IQR 0.6). When looking at the overall score of all items given to each patient, no significant difference emerged between male (median 3.3, IQR 0.6) and female panel members (median 3.1, IQR 0.4).

The ICC value for the panel was poor, 0.48 (*p* < 0.001), and per question ranged between 0.19 and 0.70, yielding poor to moderate inter-rater reliability. In Table 2, the items are listed according to the level of inter-rater reliability from best to worst. The ICC for female and male panelists was 0.44 (*p* < 0.001) and 0.48 (*p* < 0.001), respectively. The surgeons had an ICC of 0.437 (*p* < 0.001) and the non-surgeon 0.562 (*p* < 0.001).

**Table 2:** Inter-rater reliability of items.

Item	ICC	*p*-value
8, shape of breast	0.697^a^	<0.001
2, appearance of scar	0.62^a^	<0.001
10, masculinity of chest from the front	0.59^a^	<0.001
11, masculinity of chest from the side	0.563^a^	<0.001
9, size of breast	0.563^a^	<0.001
3, size of areola	0.453^b^	<0.001
1, positioning of scar	0.45^b^	<0.001
5, shape of areola	0.392^b^	<0.001
12, symmetry of chest	0.351^b^	<0.001
4, positioning of areola	0.29^b^	<0.001
7, size of nipple	0.243^b^	<0.001
6, shape of nipple	0.191^b^	<0.001

a Moderate.

b Poor. ICC, intraclass correlation coefficient.

The delay from the postoperative photographs to participants filling in the questionnaire was a median of 2 years and 5 months, with a range from 2 months to 9 years. The respondents reported a median chest score of 76 (IQR 34) and a median nipple score of 75 (IQR 37) out of 100. Table 3 presents the scores given by panelists and patients based on the surgical technique applied.

**Table 3:** Panel scores and patient-reported outcome based on surgical technique.

Technique	Overall score	Overall chest-specific items	Overall nipple-specific items	BODY-Q Chest Score	BODY-Q Nipple Score
Periareolar techniques, median (IQR)	3.4 (0.8)	3.4 (1.2)	3.3 (0.6)	67 (17)	62 (41)
Horizontal incision with pedicular NAC relocation, median (IQR)	3.3 (0.2)	3.3 (0.4)	3.1 (0.4)	93 (14)	90 (22)
Horizontal incision with free NAC graft, median (IQR)	3.1 (0.3)	3.3 (0.3)	3 (0.3)	70 (22)	65 (23)
*p*-value	0.193	0.603	0.118	0.016	0.068

IQR, interquartile range.

**Figure 3:**
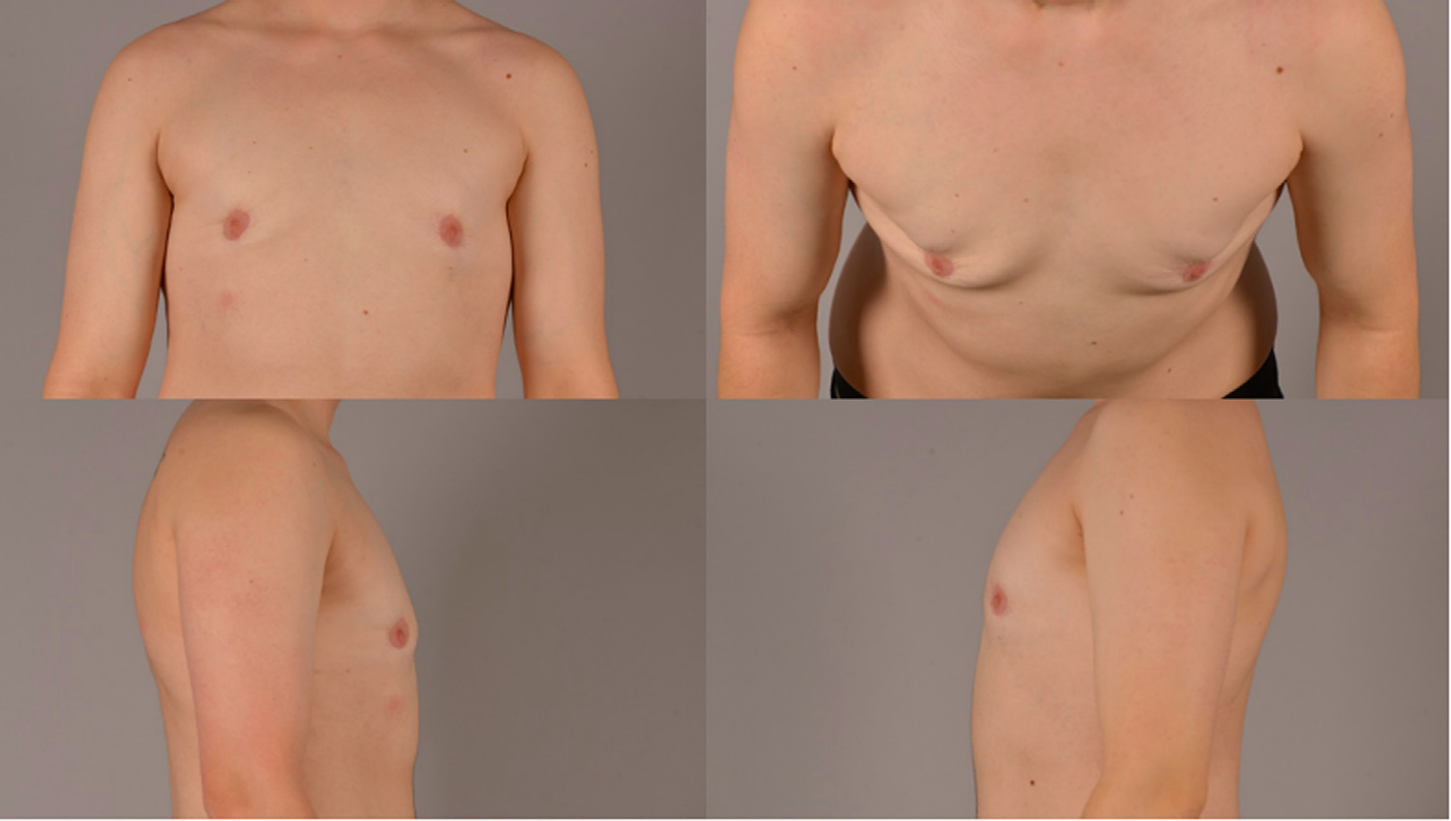
Postoperative images of patient A.

As an example, Patient A in Figure 3, was operated with periareolar technique and reported a chest score of 67 being equal to the median of chest scores among the patients operated with the periareolar technique. Furthermore, Patient A reported a nipple score of 41, which is clearly below the median, and received an overall score of 3.5 from the panel being slightly above the median.

**Figure 4:**
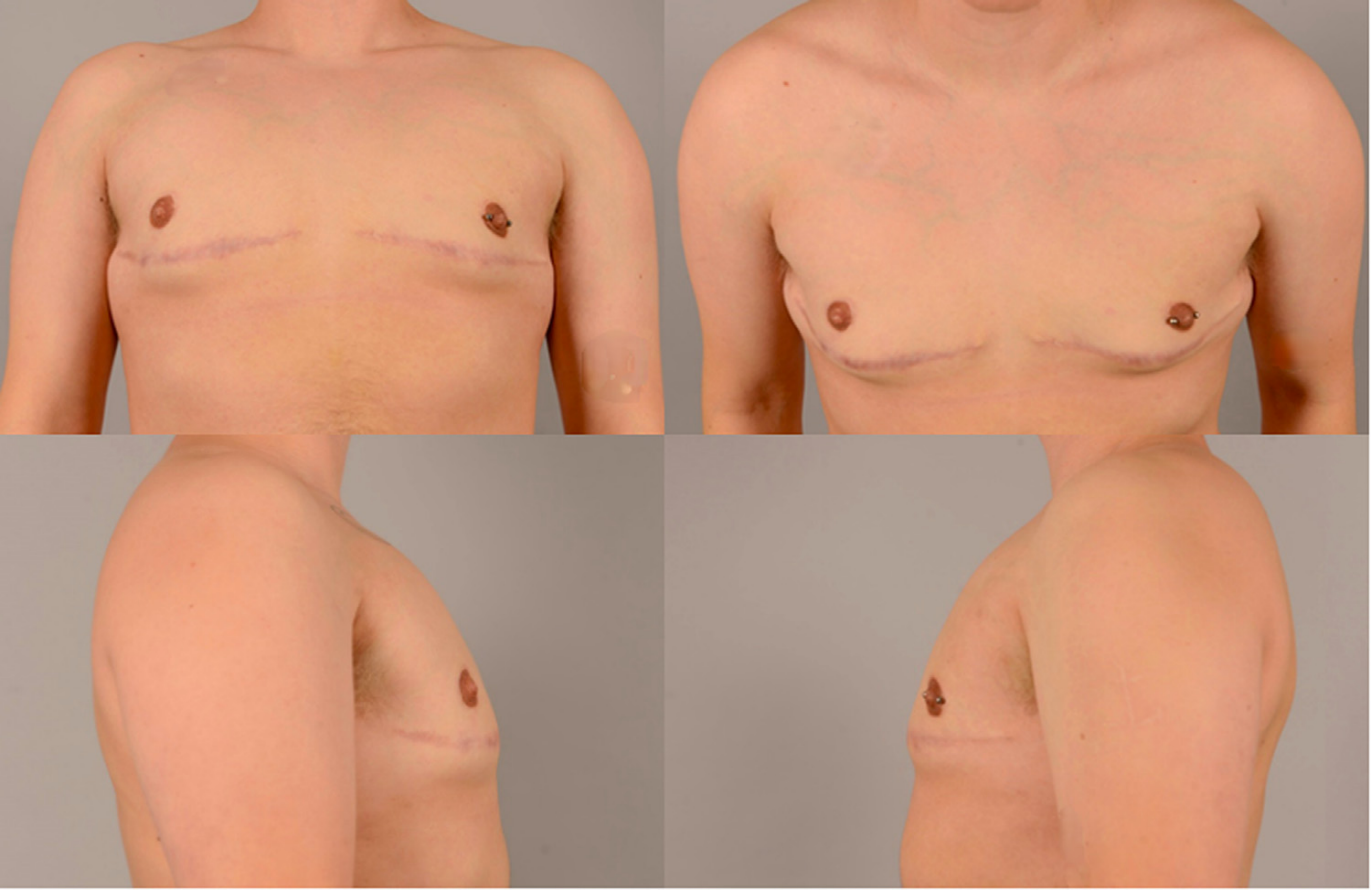
Postoperative images of patient B.

Patient B, shown in Figure 4, was operated with horizontal incision and pedicular NAC relocation. Patient B reported a chest score of 93, being equal to the median of chest scores in patients with the above-mentioned technique. The nipple score was reported as 100, being above the median, and the overall score by the panel was 3.3, being equal to the median.

When analyzing single items that were rated by the panelists and included in the BODY-Q Chest Module, the correlation coefficients between panelists and patients varied from 0.05 to 0.31, but the correlations were not statistically significant. When comparing panel and patient ratings with Kruskal-Wallis test, no statistically significant difference was detected. The items compared are listed in Table 4.

**Table 4:** List of items filled of both the panel and patients.

BODY-Q Chest Module Item 4. How masculine does your CHEST (breast area) look?
BODY-Q Chest Module Item 10. How does your naked CHEST (breast area) look in the mirror?
BODY-Q Chest Module Item 11. How do the scars from your surgery look?
BODY-Q Nipple Module Item 1 (x2). How does the shape of your nipples look?^a^
BODY-Q Nipple Module Item 2 (x2). How is the size of your nipples?^a^

a Compared with the panelists’ items regarding both areola and nipple.

**Figure 5:**
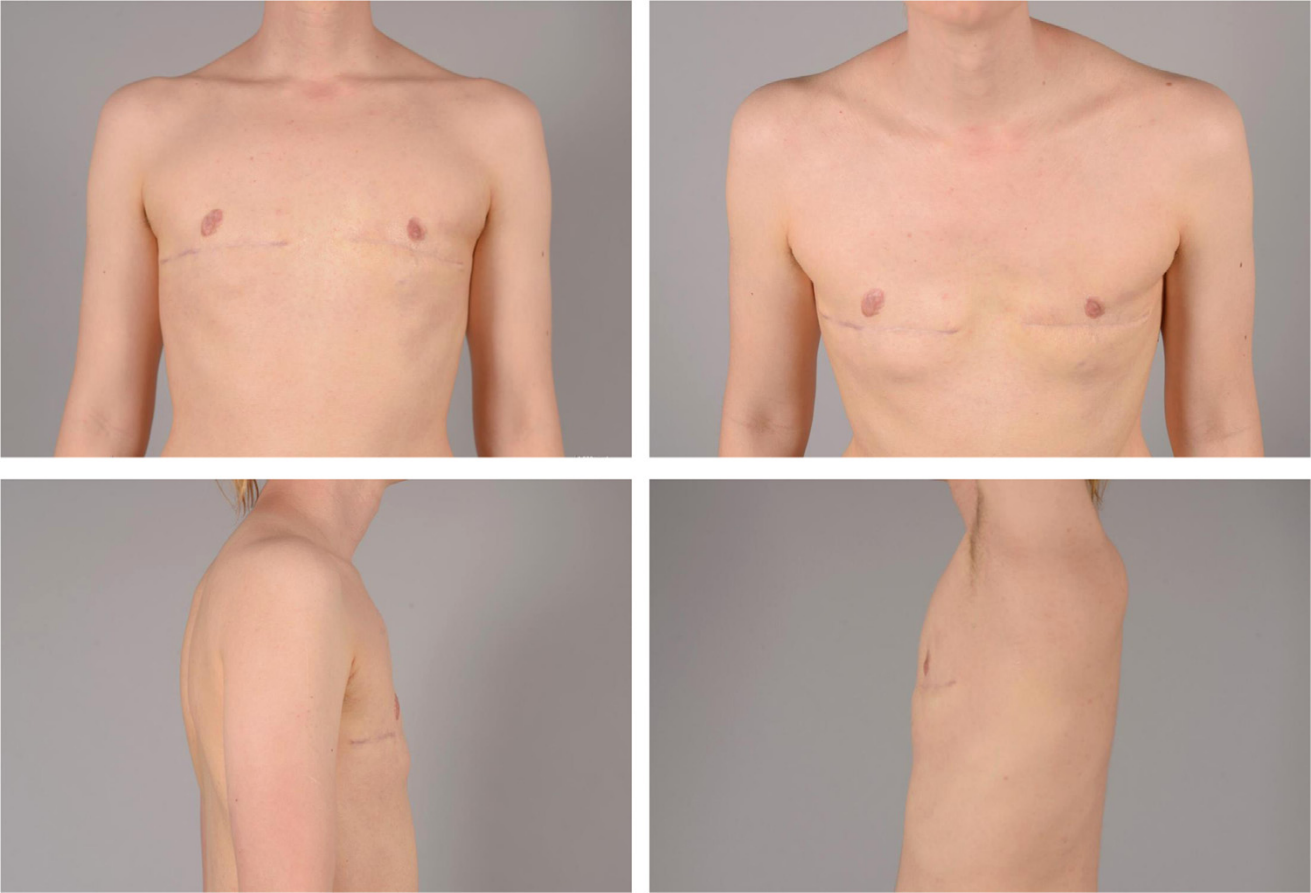
Postoperative images of patient C.

As an example, concerning patient C shown in Figure 5, the panelists’ scores varied from one to four, ranging from *very dissatisfied* to *very satisfied* regarding position of the scar, size of the areola, shape of the nipple, size of the nipple, shape of the breast, and masculinity of the chest from the side. Patient C himself reported being *satisfied* with the size of the nipples as well as the shape of the nipples. Further, Patient C reported being *very satisfied* with the masculinity of the chest from the side.

## Discussion

When evaluating postoperative esthetic results, one should be cautious regarding the benefits and limitations of different evaluation modalities. There might be discrepancy among surgeons and between patients and surgeons regarding the desirable postoperative result. If not addressed, this discrepancy might hinder reaching the optimal aesthetic result. Due to the biases of PROMs and the humane tendency to report satisfaction on self-chosen matters, we prefer to apply other esthetic outcome measures beside PROMs to gain a more comprehensive view of the outcome. Surgeons’ view of an optimal result is particularly important since it directly impacts the result of the operation.

Although scales used in previous studies vary, our panel-reported results appear similar to those in the literature, where five-point Likert scales have provided overall scores between 3.4 and 4.6, and four-point scales an average of 3.4.^15^, ^16^, ^17^, ^18^ This indicates that overall the esthetic result after chest masculinization is good.

The use of the BODY-Q Chest Module in clinical studies of gender-affirming care has been limited to date and the results have varied. However, our findings are similar to those of earlier reports, with postoperative chest scores varying between 67 and 87, while nipple scores have ranged between 58 and 90.^15^^,^ ^25^^,^ ^26^

Concerning the impact of the surgical technique, previous studies with clinician-reported outcomes have shown more favorable results with “keyhole” or concentric circular techniques than with other techniques, usually double incision with free nipple graft.^16^^,^ ^17^^,^ ^27^^,^ ^28^ Our study did not detect a significant difference between these groups. One possible explanation for the discrepancy is selection of surgical technique. In our data, periareolar techniques were used in over half of the operations, while the other studies with panel evaluation, and studies on chest masculinization in general, have more often used double incision techniques.^16^^,^ ^17^ In one study, periareolar techniques predominated, but esthetic evaluation was performed by only one surgeon, decreasing the reliability relative to a panel evaluation.^27^

Direct statistical comparison of clinician- and patient-reported outcomes has not previously been conducted, although some studies have reported both outcomes.^18^^,^ ^27^^,^ ^28^ In our data, no significant correlation nor statistical significant difference emerged between panel evaluation and patient-reported outcomes when identical items from both evaluations were inspected. This supports the theory that patients and clinicians rate esthetics differently, but not in a way where one consistently yields lower or higher scores than the other.^14^^,^ ^20^ However, since the panel and patient evaluations were performed at different time-points, the value of our finding is limited and should be interpreted with caution.

In this study, inter-rater reliability was poor. This applied especially to aspects related to the areola and the nipple. The great variation suggests a lack of consensus about the desired outcome and the optimal positioning and shaping of the nipple-areolar complex (NAC). On the other hand, regarding aspects like the shape and masculinity of the chest, a better agreement was observed. In contrast to earlier studies on esthetic evaluation, we found no significant difference between female and male raters.

In previous studies, smaller panels of three or four members have provided reliable evaluations.^9^, ^10^, ^11^ Based on these findings, our panel size should be sufficient for reliable evaluation. The panel was also gender-balanced and contained several professionals, although mostly surgeons with long experience in breast surgery, which should have added to the reliability. Based on these findings, we argue that, unfortunately, the previous knowledge on reliability of panel evaluations does not apply to chest masculinizations. We also claim that this is mainly due to a lack of consensus about the desired result among surgeons. This might be related to the fact that chest masculinizations have clearly increased only in the past decade.^29^^,^ ^30^ Furthermore, when aiming for a masculine result, several factors beside the surgery contribute to the esthetic result. For example, masculinizing hormone therapy can have a marked impact on the appearance of the chest regarding muscle mass and masculine body hair.

At the time of the study, providing chest masculinizing surgery in Finnish public healthcare was limited to binary transgender patients. However, since the proportion of non-binary patients seeking care and undergoing surgical treatment is increasing, there will be a demand on non-binary chest contouring and more individual postoperative results. This development will require new tools to communicate and measure aesthetic result after gender affirming chest surgery.

### Strengths and limitations

Due to the lack of validated clinician-reported outcome tool, the use of self-designed items with Likert scales has thus far been considered acceptable in evaluation of esthetic results.

A strength of our study is the size and composition of the panel.

An unfortunate limitation of our study is the small proportion of patients with accurate postoperative images taken. Since postoperative images are usually taken at a certain time after the primary surgery, it is likely that patients with corrective interventions had not yet reached our inclusion criteria of images needing to be taken at least 12 months after the primary surgery or in the case of secondary corrections, at least 6 months after the last correction. Therefore, patients without corrective interventions are more likely to have been included in the data.

This undoubtedly exposes our study to selection bias. The demand of preoperative images would have reduced the patient material even more and was therefore rejected. In our material periareolar technique is used more often than in other studies on the subject, which may limit the applicability of our results to other surgical techniques. The panel also lacked information on specific preferences that the patients might have had regarding the surgery, for example scar positioning. Furthermore, being a single-center and therefore a single-nation study, the applicability of our results to other contexts is limited. Another clear limitation is that the patient images and the patient-reported outcomes were obtained at different time-points. Although the postoperative wound healing should be finished after 1 year, there are several other factors that could have impacted the appearance of the chest after the images were taken, affecting panel evaluation and patient-reported results. Therefore, direct comparison of the PROM and panel evaluation might be biased.

## Conclusion

Although the reliability of panel-evaluated aesthetic result after chest masculinization is weak, both panelists and patients give good ratings on postoperative aesthetic result. However, even professionals lack a consensus on what constitutes a desirable result after chest masculinization, and neither correlation nor consistent difference was found between patient-reported results and the panel evaluation. Due to the biases of PROM, we consider the development of a clinician-reported outcome tool a crucial step in progress of chest masculinization of transgender patients. While the aim of chest masculinization is to provide the most favorable aesthetic result with discreet postoperative outcome and simultaneously manage surgical risks, the open dialogue of between the surgeon and patient is of high importance while planning for surgery. Providing the patient with real-life images of pre- and postoperative results while planning for surgery could help with shared decision-making.

## Ethical approval

The protocol of this study was approved by the Ethics Committee of Helsinki University Hospital, Finland (Decision number HUS/2711/2019). All procedures performed were in accordance with the 1964 Helsinki Declaration and its later amendments or comparable ethical standards. Informed consent was obtained from all patients who participated in the survey. A separate consent was obtained to use patient photographs.

## Funding

This study was funded by the Finnish Medical Society (Finska Läkaresällskapet) and the Musculoskeletal and Plastic Surgery Research Center Helsinki, Helsinki University Hospital, and the University of Helsinki, Finland. Open access was funded by Helsinki University Library. The funders were not involved in planning or implementing the study.

## Declaration of competing interest

The authors declare that they have no conflict of interest.
